# Prevalence and risk factors of cardiac thrombus prior to ventricular tachycardia catheter ablation in structural heart disease

**DOI:** 10.1093/europace/euac156

**Published:** 2022-11-10

**Authors:** Thomas Bonnin, Pierre Roumegou, Soumaya Sridi, Saagar Mahida, Aurélien Bustin, Josselin Duchateau, Romain Tixier, Nicolas Derval, Thomas Pambrun, Ghassen Chniti, Takamitsu Takagi, Tsukasa Kamakura, Philipp Krisai, Clementine Andre, Rémi Chauvel, Meleze Hocini, Michel Haissaguerre, Pierre Jais, Hubert Cochet, Frederic Sacher

**Affiliations:** Department of Cardiac Pacing and Electrophysiology, Hopital cardiologique du Haut-Leveque, Bordeaux University Hospital (CHU), 33604 Bordeaux, France; IHU Liryc, Electrophysiology and Heart Modeling Institute, University Bordeaux, F-33600 Bordeaux, France; Department of Cardiac Pacing and Electrophysiology, Hopital cardiologique du Haut-Leveque, Bordeaux University Hospital (CHU), 33604 Bordeaux, France; IHU Liryc, Electrophysiology and Heart Modeling Institute, University Bordeaux, F-33600 Bordeaux, France; IHU Liryc, Electrophysiology and Heart Modeling Institute, University Bordeaux, F-33600 Bordeaux, France; Department of Radiology, Bordeaux University Hospital (CHU), Bordeaux, France; IHU Liryc, Electrophysiology and Heart Modeling Institute, University Bordeaux, F-33600 Bordeaux, France; IHU Liryc, Electrophysiology and Heart Modeling Institute, University Bordeaux, F-33600 Bordeaux, France; Department of Radiology, Bordeaux University Hospital (CHU), Bordeaux, France; Department of Cardiac Pacing and Electrophysiology, Hopital cardiologique du Haut-Leveque, Bordeaux University Hospital (CHU), 33604 Bordeaux, France; IHU Liryc, Electrophysiology and Heart Modeling Institute, University Bordeaux, F-33600 Bordeaux, France; Department of Cardiac Pacing and Electrophysiology, Hopital cardiologique du Haut-Leveque, Bordeaux University Hospital (CHU), 33604 Bordeaux, France; IHU Liryc, Electrophysiology and Heart Modeling Institute, University Bordeaux, F-33600 Bordeaux, France; Department of Cardiac Pacing and Electrophysiology, Hopital cardiologique du Haut-Leveque, Bordeaux University Hospital (CHU), 33604 Bordeaux, France; IHU Liryc, Electrophysiology and Heart Modeling Institute, University Bordeaux, F-33600 Bordeaux, France; Department of Cardiac Pacing and Electrophysiology, Hopital cardiologique du Haut-Leveque, Bordeaux University Hospital (CHU), 33604 Bordeaux, France; IHU Liryc, Electrophysiology and Heart Modeling Institute, University Bordeaux, F-33600 Bordeaux, France; Department of Cardiac Pacing and Electrophysiology, Hopital cardiologique du Haut-Leveque, Bordeaux University Hospital (CHU), 33604 Bordeaux, France; IHU Liryc, Electrophysiology and Heart Modeling Institute, University Bordeaux, F-33600 Bordeaux, France; Department of Cardiac Pacing and Electrophysiology, Hopital cardiologique du Haut-Leveque, Bordeaux University Hospital (CHU), 33604 Bordeaux, France; IHU Liryc, Electrophysiology and Heart Modeling Institute, University Bordeaux, F-33600 Bordeaux, France; Department of Cardiac Pacing and Electrophysiology, Hopital cardiologique du Haut-Leveque, Bordeaux University Hospital (CHU), 33604 Bordeaux, France; IHU Liryc, Electrophysiology and Heart Modeling Institute, University Bordeaux, F-33600 Bordeaux, France; Department of Cardiac Pacing and Electrophysiology, Hopital cardiologique du Haut-Leveque, Bordeaux University Hospital (CHU), 33604 Bordeaux, France; IHU Liryc, Electrophysiology and Heart Modeling Institute, University Bordeaux, F-33600 Bordeaux, France; Department of Cardiac Pacing and Electrophysiology, Hopital cardiologique du Haut-Leveque, Bordeaux University Hospital (CHU), 33604 Bordeaux, France; IHU Liryc, Electrophysiology and Heart Modeling Institute, University Bordeaux, F-33600 Bordeaux, France; Department of Cardiac Pacing and Electrophysiology, Hopital cardiologique du Haut-Leveque, Bordeaux University Hospital (CHU), 33604 Bordeaux, France; IHU Liryc, Electrophysiology and Heart Modeling Institute, University Bordeaux, F-33600 Bordeaux, France; Department of Cardiac Pacing and Electrophysiology, Hopital cardiologique du Haut-Leveque, Bordeaux University Hospital (CHU), 33604 Bordeaux, France; IHU Liryc, Electrophysiology and Heart Modeling Institute, University Bordeaux, F-33600 Bordeaux, France; Department of Cardiac Pacing and Electrophysiology, Hopital cardiologique du Haut-Leveque, Bordeaux University Hospital (CHU), 33604 Bordeaux, France; IHU Liryc, Electrophysiology and Heart Modeling Institute, University Bordeaux, F-33600 Bordeaux, France; Department of Cardiac Pacing and Electrophysiology, Hopital cardiologique du Haut-Leveque, Bordeaux University Hospital (CHU), 33604 Bordeaux, France; IHU Liryc, Electrophysiology and Heart Modeling Institute, University Bordeaux, F-33600 Bordeaux, France; IHU Liryc, Electrophysiology and Heart Modeling Institute, University Bordeaux, F-33600 Bordeaux, France; Department of Radiology, Bordeaux University Hospital (CHU), Bordeaux, France; Department of Cardiac Pacing and Electrophysiology, Hopital cardiologique du Haut-Leveque, Bordeaux University Hospital (CHU), 33604 Bordeaux, France; IHU Liryc, Electrophysiology and Heart Modeling Institute, University Bordeaux, F-33600 Bordeaux, France

**Keywords:** Thrombus, Ventricular tachycardia, Catheter ablation, Structural heart disease

## Abstract

**Aims:**

Assess prevalence, risk factors, and management of patients with intra-cardiac thrombus referred for scar-related ventricular tachycardia (VT) ablation.

**Methods and results:**

Consecutive VT ablation referrals between January 2015 and December 2019 were reviewed (*n* = 618). Patients referred for *de novo*, scar-related VT ablation who underwent pre-procedure cardiac computed tomography (cCT) were included. We included 401 patients [61 ± 14 years; 364 male; left ventricular ejection fraction (LVEF) 40 ± 13%]; 45 patients (11%) had cardiac thrombi on cCT at 49 sites [29 LV; eight left atrial appendage (LAA); eight right ventricle (RV); four right atrial appendage]. Nine patients had pulmonary emboli. Overall predictors of cardiac thrombus included LV aneurysm [odds ratio (OR): 6.6, 95%, confidence interval (CI): 3.1–14.3], LVEF < 40% (OR: 3.3, CI: 1.5–7.3), altered RV ejection fraction (OR: 2.3, CI: 1.1–4.6), and electrical storm (OR: 2.9, CI: 1.4–6.1). Thrombus location-specific analysis identified LV aneurysm (OR: 10.9, CI: 4.3–27.7) and LVEF < 40% (OR: 9.6, CI: 2.6–35.8) as predictors of LV thrombus and arrhythmogenic right ventricular cardiomyopathy (OR: 10.6, CI: 1.2–98.4) as a predictor for RV thrombus. Left atrial appendage thrombi exclusively occurred in patients with atrial fibrillation. Ventricular tachycardia ablation was finally performed in 363 including 7 (16%) patients with thrombus but refractory electrical storm. These seven patients had tailored ablation with no embolic complications. Only one (0.3%) ablation-related embolic event occurred in the entire cohort.

**Conclusion:**

Cardiac thrombus can be identified in 11% of patients referred for scar-related VT ablation. These findings underscore the importance of systematic thrombus screening to minimize embolic risk.

## Graphical Abstract

**Graphical Abstract:**
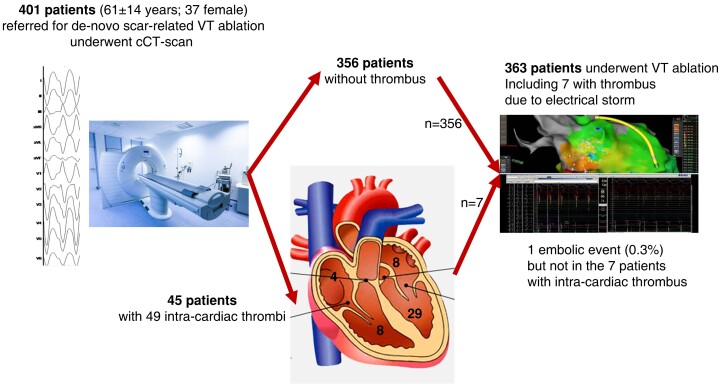

**What's new?**

- Intra-cardiac thrombus is present in up to 11% of patients referred for scar-related ventricular tachycardia (VT) ablation.
- Identification of intra-cardiac thrombus allows to adapt the strategy before scar-related VT ablation resulting in an embolic complication rate as low as 0.3%.
- Left atrial appendage (LAA) thrombus is identified in 2% of patients undergoing scar-related VT ablation. A history of atrial fibrillation is present in all of these patients with LAA thrombus.

## Introduction

Ventricular tachycardia (VT) ablation is an effective technique for the treatment of drug-refractory VT. However, VT ablation is associated with a significant risk of complications, important among that are cerebrovascular accidents due to embolic events. The incidence of stroke in patients undergoing VT ablation in the context of structural heart disease (SHD) has been reported up to 2.7%.^1^ While a number of strategies have been developed to minimize the risk of procedure-related embolic events, including peri-procedure anticoagulation, use of irrigated ablation catheters, and selective use of retrograde-aortic access, the risk of brain emboli remains high.^2^ There is therefore a need to refine strategies for the identification of patients who are at high risk of embolic events, both in terms of patient selection and for personalization of ablation strategies in high risk patients.

Intra-cardiac thrombus is a major source of embolic complications associated with VT ablation. While current guidelines recommend pre-VT ablation imaging to exclude intra-cardiac thrombus in SHD patients, due to a relative paucity of data in this specific patient population, there remains uncertainty in terms of the optimal imaging strategy and peri-procedure management in patients with intra-cardiac thrombus. The aim of this study was to assess the prevalence, risk factors, and outcome of cardiac thrombus in SHD patients who systematically underwent pre-VT ablation cardiac computed tomography (cCT).

## Methods

### Study design

In the absence of specific contraindications [glomerular filtration rate (GFR) <30 mL/min/1.73 m^2^, true allergy to contrast agent without anti-allergic preparation), pre-procedure cCT is systematically performed for all SHD patients referred for VT ablation at our institution. The aim of cCT imaging is pre-procedure definition of the arrhythmogenic substrate, intraprocedural image-integration, and exclusion of intra-cardiac thrombus. We conducted a retrospective analysis of all patients who were referred for VT ablation between January 2015 and December 2019 at Bordeaux University Hospital. Criteria for inclusion in the study were as follows: (i) patients who underwent pre-procedure cCT, (ii) patients with SHD [defined as a left ventricular (LV) ejection fraction (LVEF) < 50% and/or right ventricle (RV) dysfunction], (iii) patients planned for *de novo* VT ablation procedures (in order to eliminate the potential confounding effects of prior ablation procedures).

### Data extraction

Patients provided written informed consent for data collection in accordance with French national law (MR-004) and recommendations from the Commission Nationale de l’Informatique et des Libertés. Demographic data, data on burden of VT, results of investigations, including echocardiographic analysis and blood work (at the time of cCT imaging), procedural data, and data on embolic complications during VT ablation hospitalization were collected from electronic medical records.

### Cardiac computed tomography scan: image acquisition

Optimal hydration was performed before contrast injection particularly in patients with GFR between 30 and 50 mL/min.

**Figure 1:**
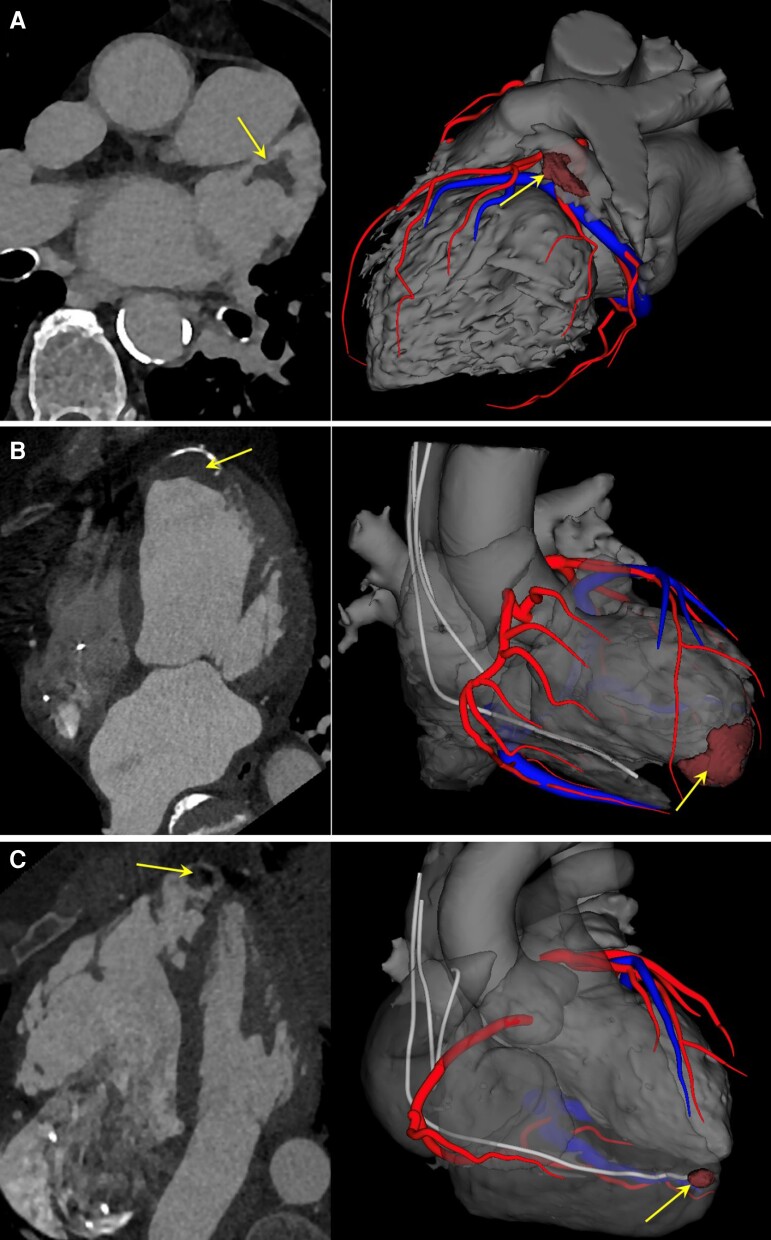
Examples of intra-cardiac thrombi on CT scan in patients referred for VT ablation. (*A*) Thrombus located in the left atrial appendage in a patient referred for VT ablation with associated history of atrial fibrillation. (*B*) Thrombus located at left ventricular apex in a patient with post-infarction VT and calcified apical aneurysm. (*C*) Thrombus located at right ventricular apex in a patient with arrhythmogenic right ventricular cardiomyopathy. Left column: contrast-enhanced CT. Right column: 3D modelling via Inheart technology. Arrows indicate thrombus location.

The standard imaging protocol involved pre-procedure cCT within 7 days of VT ablation. A dual source scanner was used for imaging (SIEMENS DEFINITION between January 2015 and March 2017; SIEMENS FORCE between March 2017 and December 2019). Contrast volume was adapted to patient weight (1 mL/kg) with a contrast media concentration of 400 mg/mL iodine if the renal function was normal and 350 mg/mL if impaired. A dual phase bolus was used with (70% administered pure at 5–6 mL/s immediately followed by 30% diluted at 50/50% with saline at 5–6 mL/s). Arterial acquisition was performed at the enhancement peak in the ascending aorta with a volume comprising the whole heart and aortic arch. Tube voltage was (80–100 kV) and images reconstructed in 0.6 mm slice thickness. Venous enhancement CT is acquired 60–90 s after contrast media injection with a volume comprising only the heart and tube voltage was lowered at 70–90 kV. This acquisition was used to rule out thrombus and imaged late to distinguish thrombus and incomplete blood mixing. All acquisitions were performed at a consistent mid-diastole phase. cCT scans were independently reviewed by two experienced radiologists blinded to the results. In order to assess intra-observer variability, repeat analysis of anonymized scans was performed by the same radiologists >6 months after the initial review. Detailed image analysis at arterial and/or venous acquisition time was performed for the presence of endocardial thrombus (in any cardiac chamber), myocardial ischaemic sequelae (wall thinning), presence of aneurysm and pulmonary embolism. Thrombus was defined as a filling defect present on both the arterial and venous acquisition time (*Figure 1*). Of note, nine patients did not have venous acquisition sequence.

### Ventricular tachycardia ablation

Procedures were performed under conscious sedation. Vascular access was obtained via the femoral vein and/or femoral artery. The LV was accessed via a transseptal (BRK Needle, Agilis sheath, St Jude Medical) and/or retrograde-aortic approach. Pericardial access, where indicated, was obtained via the subxyphoid approach (Tuohy needle, Agilis sheath, St Jude Medical). Following LV access, a 50 U/kg heparin bolus was administered intravenously, with an ACT target >250 or 300 s, depending on the case. Endocardial and/or epicardial electro-anatomical mapping was performed during sinus rhythm/paced rhythm using the Ensite (St Jude Medical), CARTO3 (Biosense Webster), or Rhythmia (Boston Scientific) mapping systems and often a multipolar high-density mapping catheter. A 3.5 mm irrigated tip catheter was used for ablation. Inducibility of VT [two sites, two drive trains (600 and 400 ms); three extrastimuli; minimum coupling interval 200 ms] was assessed in haemodynamically stable patients without thrombus on cCT. The ablation strategy involved local abnormal ventricular activity elimination or CT-guided isthmus ablation and additional conventional activation and entrainment mapping techniques for haemodynamically tolerated VT. A maximum of 50 W ablation was performed endocardially and 35 W epicardially with a temperature limit of 43°C. Of note, the presence of pulmonary embolism was considered as a contraindication to ablation.

### Statistical analysis

Statistical analysis was performed using SPSS version 17 (IBM, Armonk, NY, USA). Continuous variables with normal distribution are presented as mean ± SD. Categorical variables are presented as absolute numbers and percentages. Continuous and categorical variables were compared using the Students *t*-test and *χ*^2^ or Fishers test, respectively. Logistic regression analysis was used to perform univariable analysis. Variables with a *P*-value of <0.05 were included in multivariable analysis. A *P*-value of <0.05 was considered to be statistically significant.

## Results

### Patient characteristics

**Figure 2:**
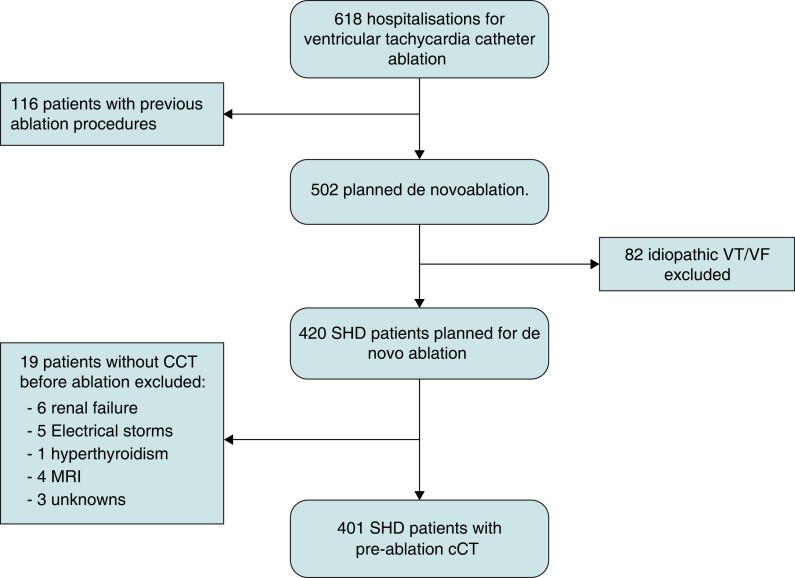
Flow chart of patients referred for VT/VF ablation. cCT, cardiac computed tomography scan; MRI, magnetic resonance imaging; RVOT, right ventricular outflow tachycardia; SHD, structural heart disease; VF, ventricular fibrillation; VT, ventricular tachycardia.

Patient selection is summarized in *Figure 2*. Between January 2015 and end of December 2019, 618 patients were referred for VT ablation with 420 (68%) *de novo* ablation in patients with SHD. Pre-procedural cCT was performed in 401/420 (95%) patient with *de novo* ablation. Baseline characteristics of the 401 included patients are summarized in *Table 1*. The average age was 61 ± 14 years. Three hundred sixty-four (91%) were male. Mean LVEF was 40 ± 13%, 59 (15%) had a left ventricular aneurysm, and 145 (36%) had RV systolic dysfunction. In terms of the underlying aetiology, 249 (62%) had ischaemic heart disease (IHD), 63 (16%) had a dilated cardiomyopathy (DCM), 38 (9%) had arrhythmogenic right ventricular cardiomyopathy (ARVC), 27 (7%) had myocarditis sequellae, 13 (3%) had congenital heart disease, and 9 (2%) had hypertrophic cardiomyopathy. Mean CHA2DS2-VASc score was 2.7 ± 1.7; 143 (36%) patients had known atrial fibrillation (AF) [91 (23%) paroxysmal; 52 (13%) persistent/permanent] and 38 (10%) patients had a previous history of stroke/TIA. One hundred fifty-five (39%) patients were on antiplatelet drugs, 98 (24%) on anticoagulant drugs, and 80 (20%) on anticoagulant and antiplatelet drugs.

**Table 1:** Baseline characteristics of the population and comparison between patients with and without cardiac thrombus

	Overall (*n* = 401)	Thrombus (*n* = 45)	No thrombus (*n* = 356)	*P*-value
Age	**61 ± 14**	**65 ± 12**	**61 ± 14**	**0.050**
Male	364 (91%)	42 (93%)	324 (90%)	0.529
BMI	27 ± 5	27 ± 5	27 ± 5	0.110
*Cardiovascular risk factors*				
ȃDiabetes mellitus	57 (14%)	7 (17%)	50 (14%)	0.785
ȃHypertension	163 (41%)	18 (42%)	145 (41%)	0.925
ȃTobacco use	102 (26%)	9 (22%)	93 (26%)	0.374
ȃHypercholesterolaemia	178 (45%)	17 (41%)	161 (45%)	0.343
ȃFamilial history of myocardial infarction	43 (11%)	3 (7%)	40 (11%)	0.351
ȃCancer	38 (9%)	2 (5%)	36 (10%)	0.221
Intra-cardiac thrombus history	**22 (5%)**	**6 (14%)**	**16 (4%)**	**0.014**
ȃStroke or TIA history	38 (9%)	5 (12%)	33 (9%)	0.691
ȃCHA2DS2-VASc	2.7 ± 1.7	3.2 ± 1.5	2.6 ± 1.7	0.025
ȃCHA2DS2-VASc ≥ 3	215 (54%)	21 (47%)	112 (31%)	0.005
*Atrial fibrillation*	143 (36%)	18 (40%)	124 (35%)	0.329
ȃPersistent/permanent	52 (13%)	8 (18%)	44 (12%)	0.308
ȃParoxysmal	91 (23%)	10 (22%)	80 (22%)	0.766
*Underlying heart disease*				
Ischaemic cardiomyopathy	**249 (62%)**	**36 (80%)**	**213 (60%)**	**0.007**
ȃDilated cardiomyopathy	63 (16%)	3 (7%)	60 (17%)	0.196
ȃMyocarditis sequelae	27 (7%)	1 (2%)	26 (7.3%)	0.061
ȃARVC	38 (9%)	5 (11%)	33 (9%)	0.691
ȃHypertrophic cardiomyopathy	9 (2%)	0	9 (2.5%)	0.281
ȃCongenital heart disease	13 (3%)	0	13 (3.6%)	0.193
ȃOther	2 (0.5%)	0	2 (0.6%)	0.614
*Biological*				
ȃHaemoglobin (g/dl)	13.6 ± 44	14 ± 1	13.6 ± 1.4	0.848
ȃHaematocrit (%)	41 ± 4	40 ± 0.7	41 ± 4	0.315
ȃPlatelet (G/L)	199 ± 65	192 ± 55	200 ± 67	0.932
ȃC-reactive protein (mg/L)	32 ± 48	21 ± 19	34 ± 51	0.637
ȃCreatinine (µmol/L)	101 ± 44	103 ± 35	101 ± 45	0.646
ȃGFR < 60 mL/min/1.73 m^2^	86 (22%)	9 (20%)	77(22%)	0.802
ȃICD	301 (75%)	31 (72%)	270 (75%)	0.841
*Antithrombotic medications*				
ȃAntiplatelet drugs	155 (39%)	21 (47%)	134 (38%)	0.851
ȃAnticoagulant drugs	98 (24%)	7 (16%)	91 (26%)	0.300
ȃAssociation	80 (20%)	9 (20%)	71 (20%)	0.957
*Treatment*				
ȃBeta-blockers	375 (94%)	42 (93%)	335 (94%)	0.958
ȃACE-inhibitors	299 (75%)	35 (78%)	266 (74%)	0.599
ȃAAD	205 (51%)	23 (51%)	183 (51%)	0.612
*Imaging*				
LVEF	**40.0 ± 12.7**	**32.9 ± 11.6**	**40.9 ± 12.6**	**<0.0001**
LVEF ≤ 40%	**235 (59%)**	**38 (84%)**	**197 (55%)**	**<0.0001**
RV systolic dysfunction	**145 (36%)**	**25 (56%)**	**120 (37%)**	**0.004**
Ischaemic sequelae	**249 (62%)**	**36 (80%)**	**213 (60%)**	**0.009**
ȃAnterior wall infarction	**97 (24%)**	**22 (49%)**	**75 (21%)**	**<0.0001**
ȃApical wall infarction	**107 (27%)**	**26 (58%)**	**81 (23%)**	**<0.0001**
ȃLateral wall infarction	41(10%)	3 (6.7%)	38 (11%)	0.403
ȃInferior wall infarction	129 (32%)	12 (27%)	117 (33%)	0.402
Aneurysm	59 (15%)	**20 (44%)**	**39 (11%)**	**<0.0001**
Electrical storm	**104 (26%)**	**21 (46.7)**	**83 (23.3)**	**0.001**

Values are mean ± SD or *n* (%). Bold values highlight parameters that are significantly different between patients with or without thrombus.

AAD, anti-arrhythmic drug; ACE, angiotensin-converting enzyme; ARVC, arrhythmogenic right ventricular cardiomyopathy; BMI, body mass index; GFR, glomerular fraction rate; ICD, implantable cardioverter defibrillator; LVEF, left ventricular ejection fraction; RV, right ventricular; TIA, transient ischaemic attack.

### Prevalence of thrombus

Forty-five (11%) patients had intra-cardiac thrombus on pre-procedure cCT. A total of 49 thrombi were identified in these 45 patients [left atrial appendage (LAA) (*n* = 8); LV (*n* = 29); right atrium (RA) (*n* = 4); and RV (*n* = 8)]. Four patients had multiple thrombus locations [LAA + RAA (*n* = 2); LAA + RV (*n* = 1); LAA + LV (*n* = 1)]. Nine (2%) had concomitant pulmonary embolism. Of note, four additional patients had a diagnosis of LAA thrombus on cCT scan performed without venous phase that was invalidated by transoesophageal echocardiography (TEE). They were therefore not included in the thrombus group. Characteristics of patients with cardiac thrombi are included in *Table 1*. Compared with patients without thrombus, patients with cardiac thrombus were older (65 ± 12 vs. 61 ± 15, *P* = 0.05), had lower LVEF (LVEF < 40%; 84 vs. 56%, *P* < 0.0001), lower right ventricle ejection fraction (RVEF) (56 vs. 38%, *P* = 0.004), a higher CHA2DS2-VASc score (3.2 ± 1.5 vs. 2.6 ± 1.7, *P* = 0.025), a higher prevalence of IHD (80 vs. 60%, *P* = 0.009), a higher prevalence of history of intra-cardiac thrombus (14 vs. 5% *P* = 0.01), ventricular aneurysm (44 vs. 11%, *P* < 0.0001), and electrical storm (47 vs. 23%, *P* = 0.001).

### Predictors of thrombus

Univariable analysis identified age, IHD, LVEF, RVEF, CHA2DS2-VASc score, LV aneurysm, and electrical storm as predictors of cardiac thrombus. On multivariable analysis, LV aneurysm [odds ratio (OR): 6.61; 95% confidence interval (CI): 3.06–14.28; *P* < 0.0001], LVEF < 40% (OR: 3.31; 95% CI: 1.50–7.28; *P* = 0.003), electrical storm (OR: 2.94; 95% CI: 1.42–6.08; *P* = 0.004), and RV systolic dysfunction (OR: 2.29; 95% CI: 1.12–4.67; *P* = 0.004) remained independent risk predictors of intra-cardiac thrombus (*Table 2*A).

**Table 2:** Univariable and multivariable analysis of (A) intra-cardiac thrombus predictors, (B) left ventricular thrombus predictors, (C) and right heart thrombus predictors

Variables	Univariable analysis		Multivariable analysis	
	*P*-value	OR (95% CI)	*P*-value	OR (95%CI)
*(A) Intra-cardiac thrombus predictors*				
LVEF < 40%	**<0.0001**	**4.38 (1.9–10.07)**	**0.003**	**3.31 (1.50–7.28)**
Electrical storm	**0.001**	**2.94 (1.42–6.08)**	**0.004**	**2.94 (1.42–6.08)**
RV dysfunction	**0.004**	**2.06 (1.13–3.85)**	**0.022**	**2.29 (1.12–4.67)**
Ischaemic sequelae	0.009	2.69 (1.26–5.76)	NS	
Aneurysm	**<0.0001**	**4.45 (2.26–8.78)**	**<0.0001**	**6.616 (3.065–14.281)**
AF	0.329	1.37 (0.73–2.57)	NS	
CHA2DS2-VASc ≥ 3	0.005	2.63 (1.32–5.26)	NS	
Anticoagulant	0.300	0.7 (0.37–1.32)	NS	
*(B) Left ventricular thrombus predictors*				
LVEF < 40%	**<0.0001**	**6.99 (2.08–23.52)**	**0.001**	**9.57 (2.56–35.79)**
CHA2DS2-VASc ≥ 3	0.001	4.59 (1.71–12.3)	NS	
Ischaemic sequelae	<0.0001	9.06 (2.12–38.7)	NS	
Aneurysm	**<0.0001**	**13.3 (5.85–30.21)**	**<0.0001**	**10.91 (4.29–27.75)**
Anticoagulant	0.361	1.42 (0.67–3.03)	NS	
*(C) Right heart thrombus predictors*				
RV dysfunction	0.001	8.85 (1.88–41.61)	0.061	4.83 (0.93–25.07)
LEVF < 40%	0.585	1.4 (0.41–4.9)	NS	
ARVC	**<0.0001**	**8.16 (2.36–28.19)**	**0.038**	**10.59 (1.13–98.64)**
CHA2DS2-VASc ≥ 3	0.711	0.8 (0.24–2.67)	NS	

Bold values highlight parameters that are significantly different between patients with or without thrombus.

AF, atrial fibrillation; CI, confidence interval; LVEF, left ventricular ejection fraction; NS, not significant; OR, odds ratio; RV, right ventricular, ARVC, arrhythmogenic right ventricular cardiomyopathy.

### Thrombus location-specific analysis

#### Left ventricular thrombus

Among the 29 patients with LV thrombus, 27 (93%) had IHD and 2 (7%) had DCM. Twenty-six (90%) had LVEF < 40%. The remaining three patients with LVEF > 40% had apical or inferior ischaemic infarcts (with aneurysm in two patients). Based on univariate analysis, LV thrombus was associated with IHD (OR: 9.06; 95% CI: 1.71–12.3; *P* = 0.001), LV aneurysm (OR: 13.3; 95% CI: 5.85–30.21; *P* < 0.0001), LVEF < 40% (OR: 6.99; 95% CI: 2.08–23.52; *P* < 0.0001), and CHA2DS2-VASc ≥3 (OR: 4.59; 95% CI: 1.71–12.3; *P* = 0.001). After multivariable analysis, LVEF < 40% (OR: 9.57; 95% CI: 2.56–35.79; *P* = 0.001) and LV aneurysm (OR: 10.91; 95% CI: 4.29–27.75; *P* < 0.0001) remained independent predictors of LV thrombus (*Table 2*B).

Among the 235 patients in the overall cohort with LVEF < 40%, thrombus at any site was identified in 36 (15%) and LV thrombus was identified in 26 (11%). Among patients with prior myocardial infarction (MI), 27/249 (11%) had LV thrombi and among patients with LV aneurysms, 17/52 (33%) had evidence of LV thrombus.

#### Left atrial appendage thrombus

All eight patients with LAA thrombus had a history of AF [paroxysmal (*n* = 4); persistent/permanent (*n* = 4)] and were already on anticoagulation. Mean LVEF was 30 ± 7% and mean CHA2DS2-VASc score was 3.6 ± 0.9. In the overall cohort, 143/401 (36%) patients had AF. Therefore, the prevalence of LAA thrombus in the patients with AF was 5.6%. Univariable analysis did not identify any variables that were significantly associated with LAA thrombus in the subset of patients with AF.

#### Right heart thrombus

Twelve thrombi were identified in the right heart [RA (*n* = 4); RV (*n* = 8)]. Based on univariable analysis, right heart thrombus was associated with ARVC and RV dysfunction. In multivariable analysis, ARVC remained an independent predictor for right heart thrombus (OR: 10.59; 95% CI: 1.13–98.64; *P* = 0.038). The results are summarized in *Table 2*C. In the subset of patients with ARVC, 5/38 (13%) had RV thrombus. All ARVC patients with RV thrombus had evidence of RV systolic dysfunction.

#### Pulmonary embolism

Pulmonary emboli were identified in 9/401 (2%) patients [ARVC (*n* = 2); myocarditis sequellae (*n* = 2); IHD (*n* = 3); DCM (*n* = 2)]. Six (67%) patients had LVEF < 40% and four (44%) RV systolic dysfunction. Only one patient (11%) was anticoagulated. As discussed above, two patients with pulmonary emboli had associated ARVC, RV dysfunction, and RV thrombus.

### Comparison with echocardiography

Twenty-seven patients with suspected cardiac thrombus on cCT scan underwent dedicated echocardiography. One patient had both LAA and LV thrombi. Concerning LV thrombi, 20 patients had a dedicated trans-thoracic echocardiography (TTE) ± contrast depending on thrombus identification. Left ventricle thrombus was only identified in 7/20 (35%) patients before contrast injection but increased to 16 (80%) after contrast use. Seven patients with diagnosis of LAA thrombus on cCT scan performed without venous phase had TEE. It confirmed LAA thrombus in three patients but invalidated it in four. The four patients without thrombus had severe LVEF impairment and all had evidence of spontaneous echo contrast on TEE. These four patients were not included in the thrombus group for analysis.

### Inter- and intra-operator reproducibility

The inter-operator reproducibility for cardiac thrombus identification on cCT scan was 90%, whereas the intra-operator reproducibility (interpretation performed >6 months after the initial one) was 96%.

### Procedural characteristics

Ablation was performed in 363 patients (356 without thrombus and 7 with thrombus). The seven patients with thrombus [LV thrombus (*n* = 4); LAA thrombus (*n* = 3)] had electrical storm and a decision was made to proceed with ablation despite the presence of thrombus. However strategy was adapted to limit embolic risk. In the four patients with LV thrombus, an epicardial approach and ablation were performed. Whereas in three patients with LAA thrombus, ablation was done via retroaortic access. Moreover no inducibility was attempted to limit the risk of electrical cardioversion. Ablation was deferred in the remaining 38 patients with thrombus. Intraprocedural electrical cardioversion was required for 116/363 (32%) patients in the overall ablation cohort. Left ventricular endocardial-only ablation was performed in 248/363 (68%). Right ventricular endocardial ablation, either alone or in combination with LV ablation, was performed in 51/363 (14%). Transseptal access was used as the sole LV-endocardial access route in 203/248 (82%) patients. A retrograde-aortic approach, either in isolation or in combination with transseptal access, was performed in 45/248 (18%). Epicardial access was used in 93/363 (26%) patients. In the seven aforementioned patients with thrombus, the LV was the target ablation site in all cases. In four patients with LV thrombus, ablation was performed via an epicardial-only approach. In three patients with LAA thrombus, ablation performed via the retrograde-aortic approach. Ventricular tachycardia inducibility was not tested in these cases to avoid the need for electrical cardioversion. No embolic complications occurred in these seven cases.

### Embolic complications

There was only one embolic complication post-ablation. The patient presented with cardiogenic shock in the context of electrical storm and required intubation and ventilation. The stroke was identified at Day 7 post-procedure, based on clinical symptoms and subsequent imaging post-extubation. The temporal relationship with the ablation procedure is therefore uncertain. Of note, there was no evidence of thrombus on pre-procedure cCT and magnetic resonance imaging in this patient.

### Follow-up of patients with intra-cardiac thrombus

Following identification of thrombus, anticoagulation was initiated in 29/45 (64%), an alternative anticoagulant was commenced in 10 (22%), antiplatelet therapy was added to anticoagulation in 4 (9%), and therapy remained unchanged in 2 (4%). Repeat imaging was performed in 24/45 (53%) after an interval of 6 ± 1.5 weeks following the index cCT scan. In 19 of these patients (79%), there was no evidence of residual thrombus. Twenty-one patients did not have further imaging [acute ablation despite thrombus (*n* = 7); death (*n* = 2); VT episodes managed without catheter ablation (*n* = 12)].

## Discussion

Cardiac thrombus remains poorly studied in patients treated with VT ablation. The main findings of the present study, which included a large cohort of patients who systematically underwent cCT scan before scar-related VT ablation, are as follows: (i) 1 in every 10 patients referred for VT ablation has evidence of intra-cardiac thrombus; (ii) when considered in combination with pulmonary emboli, which may also increase procedural risk, the prevalence of identified thrombi increases to 13%; (iii) overall, the main predictors for the presence of thrombus include LV aneurysm, LVEF < 40%, RV systolic dysfunction, and electrical storm; (iv) the rate of embolic complications using a strategy guided by pre-procedure cCT imaging is very low (0.3%). To our knowledge, only one study has evaluated the prevalence and risk factor of left ventricular thrombus in this population.^3^ They found a 6% rate of left ventricular thrombus (7% in our study) in patient with SHD before ablation. Interestingly, they have shown that ablation in these patients is safe after initiating anticoagulation.

Current consensus guidelines recommend cardiac imaging to rule out LV thrombus prior to VT ablation in patients with LV systolic dysfunction.^1^ However, the recommendations are based on expert opinion rather than systematic studies in specific populations undergoing VT ablation. The majority of studies to date has focused on patients with LV impairment in other scenarios. Among patients in the early phase post-MI, an incidence of LV thrombus of 6% has been reported, with impaired LV function, LV aneurysm, and anterior infarction as risk factors for thrombus.^4,5^ A previous CMR-based study focusing on patients with LV systolic dysfunction with multiple underling aetiologies reported an incidence of LV thrombus of 7%.^6^ Risk factors for LV thrombus included age, prior MI, and IHD. Consistent with these studies, we identified an overall prevalence of LV thrombus of 7% in our pre-VT ablation cohort, with LV aneurysm and LVEF < 40% as major predictors of thrombus. The high prevalence of LV thrombus identified in our cohort argues for systematic cCT-based screening in all patients undergoing scar-related VT ablation. In circumstances where there is limited availability of cCT, our findings indicate that LV thrombus screening is a particularly important consideration in patients with LVEF < 40% and LV aneurysm.

We reported a very low incidence of embolic complications associated with VT ablation (0.3%). Previous studies have reported procedure-related strokes in up to 2.7% of patients. Furthermore, a recent meta-analysis of 1700 patients from 18 trials found a pooled rate of embolic complications of >1%.^1,7^ There are two potential explanations for the lower embolic complication rates observed in our cohort. Firstly, systematic cCT allowed identification of intra-cardiac thrombus, which either led to procedure deferral pending thrombus resolution or, in cases where deferral was not possible, specific ablation strategies to mitigate against the embolic risk. Secondly, we used a transseptal access route in a large proportion of our cohort. Previous studies have reported a higher risk of embolic complication associated with retrograde-aortic access.^2^ We believe that our personalized approach based on cCT-based risk stratification led to a significant reduction in risk.

After LV, the second most frequent thrombus location in our cohort was the LAA. Previous studies have reported a prevalence of LAA thrombus of 2–4% among patients with non-paroxysmal AF, despite systematic anticoagulation. In our cohort, LAA thrombi were exclusively identified in patients with AF. When considering only the subset of patients with AF, the prevalence of LAA thrombus was 6.3%, despite systematic anticoagulation. The relatively higher prevalence may be explained by the CHA2DS2-VASc score in our cohort. The prevalence of LAA thrombus has potentially important implications in terms of choice of access route. In our institution, the majority of endocardial ablation procedures is performed via the transseptal access route. On one hand, this approach is predicted to reduce the embolic risk associated with aortic instrumentation, however, on the other hand, given the prevalence of LAA thrombus in AF patients, transseptal access maybe associated with an increased embolic risk. The risk maybe augmented further by the fact that up to a third of patients require cardioversion during VT ablation procedure in our study. Overall, our findings provide further support to the argument that systematic screening for LAA thrombus should be performed among AF patients undergoing VT ablation, irrespective of anticoagulation status.

Systematic screening for right heart thrombus is rarely performed prior to VT ablation, despite the fact that thrombosis is a recognized complication associated with right heart cardiomyopathies, particularly ARVC. Previous studies among ARVC cohorts have reported a prevalence of right heart thrombi of up to 4%.^8^ In the present study, we identified right heart thrombi in 13% of patients with ARVC. The higher prevalence could be explained by the fact that patients requiring VT ablation may have more advanced disease and consequently more severe RV impairment. Pulmonary embolism is a potentially fatal complication of right heart thrombus, particularly in the context of severe RV dysfunction and the inherent haemodynamic changes associated with VT ablation procedures. Our findings indicate that among patients with significant RV dysfunction, systematic screening for RV thrombus and pulmonary emboli could have an important impact on procedure-related risk.

The 2019 consensus guidelines do not specify the optimal imaging modality to exclude intra-cardiac thrombus.^1^ Trans-thoracic echocardiography is included as a potential imaging modality, however TTE is limited by significant inter-observer variability and is also reliant on optimal imaging windows. Furthermore, previous studies have reported a sensitivity of 30% with non-contrast TTE and 60% with contrast TTE.^9^ There is a relative paucity of data on cCT for LV thrombus screening. Based on limited data, sensitivity of >90% has been reported.^10^ While our study was not specifically designed to evaluate the accuracy of TTE, consistent with the aforementioned studies, TTE was associated with a sensitivity of only 35% compared with cCT. These findings indicate that TTE is not an optimal imaging modality for detection of LV thrombus in the absence of systemic use of contrast. In the atrium on the other hand, we demonstrate a comparable sensitivity between cCT and TEE for detection of LAA thrombus. However, a potential limitation of cCT in our study and others is the difficulty in distinguishing between circulatory stasis and thrombus in a minority of patients with severe LV function, which can reduce specificity and predictive positive value^11^ particularly in the absence of venous acquisition time in addition to arterial acquisition time. Therefore, when using cCT scan to rule out intra-cardiac thrombus, arterial and venous acquisition time are mandatory. Overall, cCT reliably excludes ventricular and left atrial thrombi, as well as right heart thrombus and pulmonary embolism. It may also help for VT ablation with channel/scar identification as well as critical structure (coronary artery, phrenic nerve, …) localization when imported in 3D mapping system. It has therefore the potential to simplify scar-related VT ablation and limit procedure complications.

### Limitations

This study has the inherent limitations of a retrospective single centre study performed in a highly experienced, high volume centre. Cardiac computed tomography was used as the sole imaging modality in the majority of our cohort, and has therefore not been systematically compared with other imaging modalities. However several studies^10,11^ have shown cCT efficacy compared with other imaging modality to rule out intra-cardiac thrombus.

Women represents only 9% of the patients included in this study, which is mainly driven by the IHD substrate and is in phase with the proportion found in the different studies on scar-related VT ablation.

## Conclusion

The present study identified intra-cardiac thrombi in more than one in every 10 patients undergoing VT ablation. Our findings underscore the importance of detailed systematic imaging for cardiac thrombus prior to VT ablation and tailored image-based strategies to minimize complications including embolic risk.

## Data Availability

The data underlying this article cannot be shared publicly due to RGPD policy as well as patient authorization via MR 004 regulation. The data will be shared on reasonable request to the corresponding author.
